# Efficiency in chronic illness care coordination: public-private collaboration models vs. traditional management

**DOI:** 10.1186/s12913-020-05894-z

**Published:** 2020-11-16

**Authors:** José Luis Franco Miguel, Carmen Fullana Belda, José Manuel Cordero Ferrera, Cristina Polo, Roberto Nuño-Solinís

**Affiliations:** 1 Comillas Pontifical University, C/ Alberto Aguilera 23, 28015 Madrid, Spain; 2 University of Extremadura, Badajoz, Spain; 3 University of Deusto, Bilbao, Spain

**Keywords:** Care coordination, Efficiency, Hospital management, Data envelopment analysis, Chronic disease management

## Abstract

**Background:**

The aim of this paper is to analyze the differences in the coordination of chronic illness care between the different public hospital management models coexisting in the Spanish region of Madrid (25 hospitals) during the period 2013–2017.

**Methods:**

The performance of hospitals might be affected by the characteristics of the population they serve and, therefore, this information should be taken into account when estimating efficiency measures. For this purpose, we apply the nonparametric Data Envelopment Analysis (DEA) conditioned to some contextual variables and adapted to a dynamic framework, so that we can assess hospitals during a five-year period. The outputs considered are preventable hospitalizations, readmissions for heart failure and readmissions for chronic obstructive pulmonary disease, whereas the inputs considered are the number of beds, personnel (physicians and other healthcare professionals) and total expenditure on goods and services.

**Results:**

The results suggest that the level of efficiency demonstrated by the public-private collaboration models of hospital management is higher than traditionally managed hospitals throughout the analyzed period. Nevertheless, we notice that efficiency differences among hospitals are significantly reduced when contextual factors were taken into account.

**Conclusions:**

Hospitals managed under public-private collaboration models are more efficient than those under traditional management in terms of chronic illness care coordination, being this difference attributable to more agile and flexible management under the collaborative models.

**Supplementary Information:**

**Supplementary information** accompanies this paper at 10.1186/s12913-020-05894-z.

## Background

The welfare state has led to the establishment of very complex health systems with high care fragmentation [1]. In this context, one of the main priorities worldwide is improving the quality, efficiency and coordination of the treatment for chronic conditions. In the specific case of the Spanish health system, this task is assumed by the regional governments, as they are responsible for the provision and management of healthcare services. In the last decade they have begun to implement different coordination of care strategies, primarily focused on integrated care innovations for patients with chronic conditions [2].

This study is focused on the region of Madrid (Autonomous Community of Madrid) and its healthcare strategy for patients with chronic conditions launched in 2012 [3]. Along with this strategy, innovative models, such as private financing initiatives (PFI) and public-private partnerships (PPP), began to operate in the region’s hospital system, together with public hospitals and other healthcare entities, such as foundations and state-owned hospital enterprises, in order to improve the efficiency of healthcare delivery [4, 5]. The coexistence of these different management models, whose main characteristics are summarized in Table 1, under the common regulatory framework of the Spanish NHS makes this region an interesting case study.

**Table 1:** Main characteristics of the different hospital management schemes in Madrid

	Traditional Management	PFI Management	PPP Management	Other Forms of Management
Legal status	No	Yes	Yes	Yes
Own treasury	No	Yes	Yes	Yes
Public control	Previous	A posteriori	A posteriori	Yes
Organisational structure	Organisation by specialties	Organisation for management areas	Organisation for management areas	Organisation for management areas
Own debt	No	Yes	Yes	Yes
Human resources management	Statutory regime	Labor legislation	Labour legislation	Labour legislation
Staff recruiting system	Set by SERMAS	Flexible	Flexible	Flexible
Outsourcing health services	No	No	Yes	Yes in singular concert
Outsourcing non health services	No	Yes	Yes	Yes in singular concert
Ownership of the hospital	Public	Public	Public	Public/Singular concert
Management system	Public	Non-health care services private concession	Private concession of all services	Public/Singular concert
Concession period	Not applicable	30 years	30 years	Not applicable
Number of hospitals	11	7	3	4
Potential patients assigned	3.778.465	1.546.703	416.456	908.176
Number of beds	9.676	1.641	677	2.010

Source: own elaboration

The reason to introduce these new public-private collaboration models (PFI and PPP models) is the belief that they should contribute to increasing the efficiency in the healthcare sector. However, the empirical evidence on this topic is mixed [6] and in many settings, the introduction of these new models has been the subject of widespread controversy [7]. The particular case of Madrid is not an exception, since public-private partnerships in healthcare have received multiple criticisms [8].

In this context, we focus on analyzing care coordination at the hospital level because the region of Madrid implemented in 2010 a system in which patients could freely choose their doctors and hospitals, including specialists. This initiative resulted in the unified health district of the region. Within this new framework, there is no link between a specific hospital and its primary care providers since each hospital can receive patients from any primary care provider operating in the region. This arrangement promotes the competition among hospitals to attract a greater number of patients. Within these new competitive rules, it is relevant to study if this arrangement is an efficient way of coordinating care for chronic patients. In order to address this subject we apply nonparametric frontier methods as in other previous studies focused on measuring efficiency performance.

The use of these methods is very common in the literature as shown recently in a comprehensive systematic review focused on hospitals [9]. Among them, the most frequent option is the Data Envelopment Analysis (DEA), which has been previously used in the Spanish context to analyze different models of hospital ownership and management [10–12]. The results obtained are mixed. For instance, the most recent study [10] concluded that PPP formulas favored hospital efficiency, while in other study the findings were inconclusive [11]. Likewise, in a previous empirical analysis focused on hospitals belonging to the Madrid Regional Health Service, the results suggest that there are no significant differences in terms of technical efficiency between public hospitals and those adopting new management formulas [12]. However, none of these studies analyzed outputs specifically related to the coordinated organization of care for chronic diseases, which has been identified as a priority in the health policy agenda [13]. Therefore, one of the main innovations of the present work relies on the consideration of quality of care output variables, which has only been used previously in empirical studies focusing on primary care providers [14].

Moreover, most previous studies have the common limitation of not taking into account the characteristics of the population served by each hospital in the assessment of their performance. Thus, one of the contributions of the present work is that we estimate efficiency measures incorporating the influence of those characteristics, represented by the age and the case mix of the population, in a nonparametric framework. For that purpose, we apply the nonparametric conditional model proposed by Daraio and Simar [15–17] in a dynamic framework, since we have panel data available, following the extension developed by Mastromarco and Simar [18]. This approach presents several advantages over other conventional nonparametric approaches that have been traditionally applied in this framework such as the two-stage approaches. Furthermore, this approach has been rarely applied in the health care setting [19–21] and even less to analyze the performance of hospitals, although there are a few exceptions [22, 23]. Therefore, from a methodological point of view, this article also has a clear innovative character.

This study tries to provide new empirical evidence that can be useful to highlight the current debate about the use of different forms of management in the health sector. In particular, we assess whether public-private collaboration formulas are efficient alternatives to the traditional existing management models in a framework where the coordination of chronic illness has a great relevance. In this sense, the results of our empirical study would be relevant both for policy makers and healthcare managers.

## Methods

Our empirical analysis of the performance of hospitals focuses on evaluating their technical efficiency levels in relative terms [24]. For that purpose, we rely on a nonparametric approach such as the DEA, which has been extensively used to assess the performance of a multitude of units operating in the public sector, including healthcare [25–27]. One of the major reasons for using this method is that it requires no or very limited assumptions to be made about the properties of the production technology. Moreover, this method can be easily and simultaneously adapted to processes involving not only a range of inputs but also several output dimensions, as it is common in the case of hospitals [28–30].

DEA uses linear programming to build an efficient production frontier from best-practice units, so the inefficiency of the rest of the units can be measured as the distance from the boundary. The efficiency score is defined a relative measure on a scale between 0 and 1, where the value 1 means that a hospital is placed at the frontier (efficient) and all values below 1 indicate that the hospital is inefficient (below the frontier), thus the level of inefficiency can be measured as the difference between the efficiency score and 1.

The standard formulation of the program can take several forms, depending on whether we are interested in reducing inputs or increasing output values. In this study, we have opted for an output orientation, because hospital managers have a limited capacity to reduce the resources used, at least in the short-term; thus, they can only focus on maximizing the level of outputs given the available resources [31, 32]. Likewise, we assume variable returns to scale (VRS), since in our sample, there are production units with very different sizes, and as a result, there might be different scales of production.

An important issue that must be taken into account when evaluating the efficiency of hospitals is that their performance is frequently influenced by contextual factors, which are beyond the scope of hospital management. For instance, the production possibilities of certain hospitals might be affected by the characteristics of the population covered (older age or health status of patients represented by a morbidity index based on their case-mix). Hence, those contextual or exogenous factors (Z) should be taken into account in the efficiency estimation [33]. In the literature, we can find different approaches to incorporate them into the efficiency analysis [34, 35]. However, most of them rely on the separability condition between the input-output space and the space of the external factors, i.e., assuming that these factors have no influence on the attainable set, affecting only the probability of efficiency, which may not hold in most cases [36].

In this study, we adopt the nonparametric conditional approach developed by Daraio and Simar [15] based on the previous work by Cazals et al. [37], which avoids the restrictive separability assumption required by traditional approaches and incorporates the effect of exogenous variables directly into the estimation of efficiency scores. In the following lines, we provide a nontechnical description of this methodology to facilitate the interpretation of results. Additionally, we also provide a more detailed explanation of this approach and its main computational issues in the Additional file 1.

The conditional approach is based on a probabilistic formulation of the production process, which allows for the accounting of the variables in the efficiency estimation by conditioning the production process to a given value of Z = z. For example, if the contextual variable Z represents a morbidity index of patients served by each hospital, where higher values represent a more severe health condition, this approach estimates efficiency measures by comparing the performance of each hospital with other hospitals treating patients with similar characteristics, i.e., those having Z values within a determined range defined by an interval (in our framework this interval is determined by the so-called “bandwidth”), which we estimate using the procedure suggested by Badin et al. [38]. The mathematical formulation of this approach is provided in the Additional file 1.

In addition, because longitudinal data are available, we have adapted this approach to a dynamic framework by considering the time factor *(t)* as an additional contextual variable following the model proposed by Mastromarco and Simar [18]. In this framework, we analyze the pooled dataset, i.e., a single frontier is constructed and hospitals are simultaneously compared with one another and across time; thus, we implicitly assume that there are no changes in the production technology between periods. However, it is possible that the efficiency level of hospitals in a period might depend on the efficiency in other periods.

To examine the potential influence of conditional factors (contextual variables and time) on the attainable frontier, we analyze the observed values of the ratio of the conditional efficiency scores over the unconditional scores (those estimated without considering the effect of Z variables) against Z [15–17]. In an output-oriented conditional model, an increasing trend in the ratio denotes a favorable effect of the contextual variable on the efficient frontier, since it operates as an extra input that is freely available. In contrast, a downward trend means that the effect of the contextual variable on the efficient frontier is unfavorable because it assumes the role of an extra undesirable output to be produced, which requires the use of more inputs.

Finally, it is also noteworthy that this methodological approach allows us to investigate the statistical significance of Z in explaining the variations of efficiency levels. For that purpose, we use the bootstrap test proposed by Racine [39]. This procedure roughly consists of a nonparametric regression of the ratios on the exogenous variables, which can be interpreted as the nonparametric equivalent of the standard t-tests used in ordinary least squares regression models [40]. Accordingly, each of the *p*-values will determine whether the *Z* variables have a significant influence or not.

## Data and variables

The target population is composed of 25 Madrid-region public health service hospitals that meet the requirements for being classified as general hospitals (hospitals that treat patients with any kind of condition and have general medicine, surgery, obstetrics and gynecology and pediatrics departments). Specialized hospitals (e.g., mental health institutions) and long- and medium-stay hospitals (hospitals that treat patients who, due to chronic processes or a low level of functional independence for daily living activities, need generally uncomplicated healthcare that cannot be provided at home and requires a prolonged hospital stay) are excluded from the study. Likewise, hospitals providing contract-based care only for specific activities for Madrid Health System patients are also excluded, because they do not assume responsibility for the provision of all the health services covered by the Spanish National Health System service portfolio for the population.

The sample includes hospitals under four different types of management: (i) eleven traditional public hospitals; (ii) seven hospitals managed according to the PFI model; (iii) three hospitals operating according to the PPP management model; (iv) four hospitals managed with different options.

The data used in this research were gathered from annual reports published by all the hospitals belonging to SERMAS over a five-year period (from 2013 to 2017) [41]. The reports provide data on the installed material, human resources and the activities conducted, which are collected through a specific computer application that guarantees that all data are gathered following homogeneous criteria, facilitating comparability across hospitals.

Table 2 displays the definition of all the inputs and outputs used in the analysis. As inputs we include the number of full-time equivalent employees differentiating between two professional categories (physicians and other healthcare professionals), the number of beds as a measure of hospital fixed assets and the total expenditure on goods and services (including pharmaceutical expenditure) as a proxy for current expenditure [42]. This selection is based on previous literature and, more specifically, on the guidelines established by Ozcan [43] and Kohl et al. [9] in their reviews on hospitals efficiency assessments. At this point, we should clarify that we prefer to use data about employees rather than personnel costs in order to avoid potential distortions in the estimation of technical efficiency measures due to the existence of divergences in salary levels between some professionals [44]. Those divergences should not be attributable to hospitals, since staff salaries are established by the SERMAS.

**Table 2:** Definition of inputs and outputs used and descriptive statistics

	Name	Definition
**INPUTS**	Beds	Total number of hospitalization beds available at each hospital
	Medical staff	Total number of physicians working at each hospital
	Non-medical staff	Total number of health care (non-medical) professionals working at each hospital
	Expenditure on goods and services	Actual expenditure on the purchase of goods and services (in Euro)
**OUTPUTS**	Preventable hospitalizations	Number of hospital admissions that could have been avoided
	Readmissions for COPD	Number of hospital readmissions after discharge due to a COPD diagnostic process
	Readmissions for heart failure	Number of hospital readmissions after discharge due to a heart failure diagnostic process
**CONTEXTUAL VARIABLES**	% Population > 65	Percentage of population above 65 years
	Morbidity index	Quotient between the annual number of hospital discharges per main diagnosis of people residing in the Community of Madrid and the corresponding number of people-years, estimated as the average population residing in the Community of Madrid.

Source: own elaboration

The selected outputs are related to three priority diseases highlighted in the Chronicity Strategy of Madrid’s regional government, which are: chronic obstructive pulmonary disease (COPD), diabetes type 2 and heart failure [45]. The outputs were selected while also considering proxy indicators related to care coordination, such as preventable hospitalizations (including acute complications from type 2 diabetes), and 30-day readmissions for COPD and heart failure [46]. All the selected outputs are undesirable (the lower their number is, the better the quality of service provided by the hospital will be); thus, original values need to be transformed. Among the different methodological options that can be used to address this problem (see Cordero et al. [19] for details), we have followed the method proposed by Seiford and Zhu [47] which consists of transforming the original values of this variable by multiplying them by − 1 and adding a sufficiently large parameter. In our case, we selected a value slightly higher than the maximum value for each output variable. It is noteworthy that this transformation process is only valid when variable returns to scale are assumed [48]; thus, this is an additional reason to conduct our empirical analysis under VRS.

We have also selected two contextual variables in accordance with previous literature. In particular, we use the percentage of the population above 65 years old living in the hospital’s area of influence and a morbidity index based on a case-mix system. The calculation is performed as a quotient between the annual number of hospital discharges per main diagnosis of people residing in the region of Madrid and the average age of the total population in the same region [41].

Table 3 shows the descriptive statistics for all the variables included in the analysis (inputs, outputs and contextual variables) for each year. Here we can observe that the average values of all inputs remained fairly constant over the first three years of the period under analysis. However, while the number of beds was slightly reduced in the last two years, medical staff and expenditures on goods and services experienced a remarkable increase in those years. More oscillations are observed in the outputs, although the general trend throughout the evaluated period is upwards. Finally, as expected, the socio-demographic variables selected as contextual indicators have fairly similar values in all years.

**Table 3:** Descriptive statistics

		2013				2014				2015				2016				2017			
		Mean	SD	Min	Max	Mean	SD	Min	Max	Mean	SD	Min	Max	Mean	SD	Min	Max	Mean	SD	Min	Max
**INPUTS**	Beds	534.72	403.93	91	1671	530.92	409.12	91	1671	527.64	403.75	91	1671	507.04	383.82	91	1525	503.92	363.77	91	1351
	Medical staff	441.32	285.18	97	1006	452.16	298.02	97	1110	452.96	313.69	94	1086	623.12	482.50	103	1693	648.16	477.76	110	1702
	Non-medical staff	1319.56	1088.70	248	4236	1271.52	988.01	201	3698	1299.88	1025.70	243	3780	1344.6	1082.27	260	3831	1381.68	1075.04	260	3856
	Expenditure on goods and services	79,487	69,147	8526	229,411	76,025	62,910	8146	215,834	75,374	60,930	8078	212,164	86,427	76,602	8950	61,569	86,129	69,373	9061	238,173
**OUTPUTS**	Preventable hospitalizations	1932	1205	529	5162	1993	1247	523	5456	2127	1291	626	5607	2232	1289	675	5707	2132	1292	626	5607
	Readmissions for COPD	69.62	43.58	15	218	63.84	37.1	16	188	71.68	38.13	20	196	73.28	37.21	26	209	72.14	37.95	35	284
	Readmissions for heart failure	90.82	69.03	8	279	74.32	43.68	25	241	94.22	82.43	14	414	91.35	80.52	23	418	85.68	77.48	30	421
**CONTEXTUAL** **VARIABLES**	% Population > 65	15.08	4.45	7.48	23.01	15.50	4.47	8.01	24.13	15.80	4.35	8.29	24.20	16.07	4.20	9.17	24.19	16.04	4.86	5.24	27.54
	Morbidity index	7.35	2.70	2.86	15.02	7.53	2.63	3.42	15.15	7.61	2.62	3.40	15.45	7.63	2.64	3.49	15.35	7.66	2.66	3.35	14.87

Source: own elaboration

## Results

Table 4 shows, for each category of hospitals, the mean efficiency scores estimated using an unconditional DEA model, i.e., considering only data about inputs and outputs for all the analyzed hospitals over the whole evaluated period (2013–2017). These values show that the hospitals adopting the new management models (private finance initiative and public-private partnership) achieve higher efficiency scores (with a mean value of 0.87 and 0.88 respectively) than the traditionally managed hospitals (0.77). This evidence is corroborated by the *p*-values yielded by the Kruskal-Wallis tests, which show that there are significant differences between these two groups at a 95% confidence level. This result is consistent with other empirical studies in which privatization schemes are associated with higher efficiency levels [21, 49]. Nevertheless, when we account for the characteristics of population and time in a conditional model, the differences between the management models are much smaller, as shown by the mean values reported in Table 5 (PFI and PPP present mean values of 0.94 and 0.96, respectively, while those with public management has a mean value of 0.93). Even so, the divergences between them are still significant according to the values of the Kruskal-Wallis tests reported in the Table.

**Table 4:** Mean efficiency scores of each category of management by year (unconditional model)

Hospital	2013	2014	2015	2016	2017	Average
**PFI**	**0.891**	**0.874**	**0.869**	**0.849**	**0.873**	**0.871**
	(0.080)	(0.084)	(0.070)	(0.079)	(0.074)	(0.076)
**PPP**	**0.934**	**0.932**	**0.849**	**0.859**	**0.820**	**0.879**
	(0.023)	(0.034)	(0.130)	(0.062)	(0.074)	(0.046)
**Public management**	**0.834**	**0.742**	**0.767**	**0.741**	**0.767**	**0.770**
	(0.100)	(0.129)	(0.121)	(0.112)	(0.116)	(0.103)
**Others**	**0.771**	**0.824**	**0.759**	**0.726**	**0.752**	**0.766**
	(0.078)	(0.128)	(0.091)	0.108)	(0.102)	(0.098)
**TOTAL**	**0.8517**	**0.8149**	**0.8038**	**0.7832**	**0.8004**	**0.8108**
	(0.096)	(0.127)	(0.110)	(0.109)	(0.106)	(0.100)
**Kruskal-Wallis test**	**0.048**	**0.044**	**0.049**	**0.041**	**0.046**	**0.047**

Source: own elaboration

Note: Standard deviation in brackets

**Table 5:** Mean efficiency scores of each category of management by year (conditional model)

Hospital	2013	2014	2015	2016	2017	Average
**PFI**	**0.915**	**0.946**	**0.937**	**0.948**	**0.953**	**0.940**
	(0.065)	(0.050)	(0.051)	(0.049)	(0.041)	(0.046)
**PPP**	**0.971**	**0.972**	**0.939**	**0.978**	**0.945**	**0.961**
	(0.036)	(0.034)	(0.054)	(0.038)	(0.039)	(0.033)
**Public management**	**0.913**	**0.913**	**0.946**	**0.951**	**0.959**	**0.936**
	(0.087)	(0.090)	(0.051)	(0.044)	(0.041)	(0.039)
**Others**	**0.844**	**0.915**	**0.974**	**0.923**	**0.943**	**0.920**
	(0.071)	(0.091)	(0.034)	(0.082)	(0.070)	(0.059)
**TOTAL**	**0.909**	**0.929**	**0.947**	**0.949**	**0.953**	**0.937**
	(0.078)	(0.075)	(0.048)	(0.051)	(0.044)	(0.043)
**Kruskal-Wallis test**	**0.043**	**0.048**	**0.046**	**0.045**	**0.042**	**0.040**

Source: own elaboration

Note: Standard deviation in brackets

**Fig. 1:**
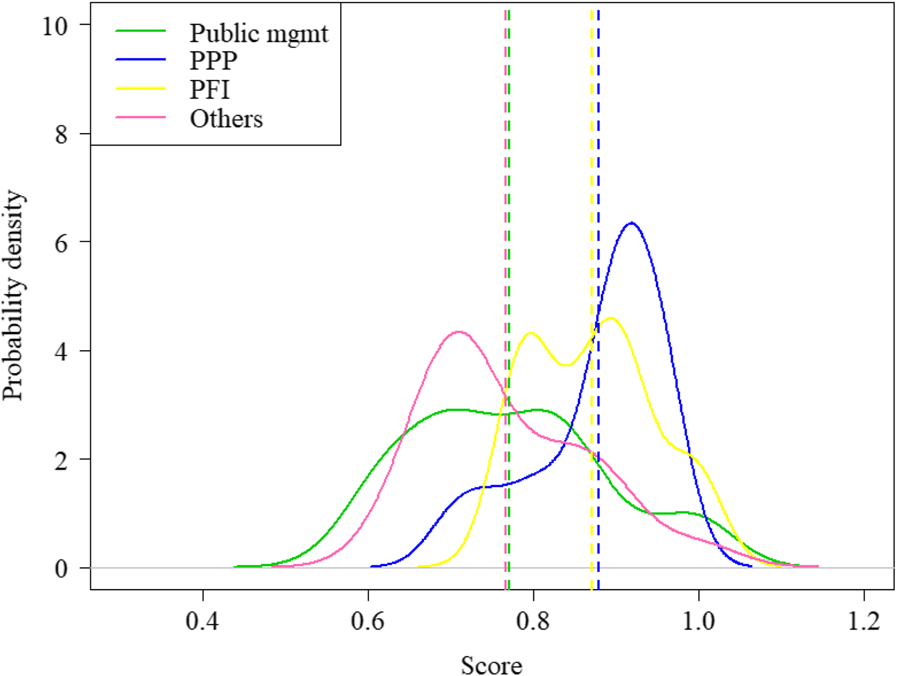
Density distribution of efficiency scores by category of management (unconditional model)

**Fig. 2:**
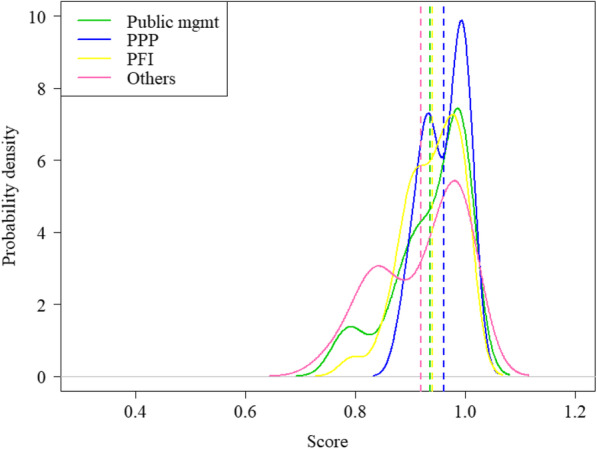
Density distribution of efficiency scores by category of management (conditional model)

Moreover, given that mean values might hide some relevant information, we also consider an additional tool that allows for a fuller view of the distributions of the estimated measures of performance distinguishing between different types of management. Specifically, Figs. 1 and 2 display the estimated distributions of both efficiency estimates, respectively, calculated using nonparametric kernel density methods. In the first figure, we notice that the distribution of PPP and PFI hospitals is quite different despite having a similar value. Thus, we observe that there is greater variability in the efficiency scores obtained by PPP hospitals, with a high proportion of units positioned to the right of the average value (represented by the vertical dashed line) and other several units well below that mean value. On the contrary, the distribution of hospitals managed by private finance initiatives is less extensive, and most of them are located around two modes. In the second figure, all the distributions of conditional efficiencies are concentrated on higher levels of efficiency, since the reference set for comparison is further reduced as this only includes units with similar contextual conditions. Therefore, it is quite predictable that many hospitals would increase their efficiency levels when we account for the context and dynamic effects. Nevertheless, we observe that the new management models are above the rest, especially the public-private partnership.

**Fig. 3:**
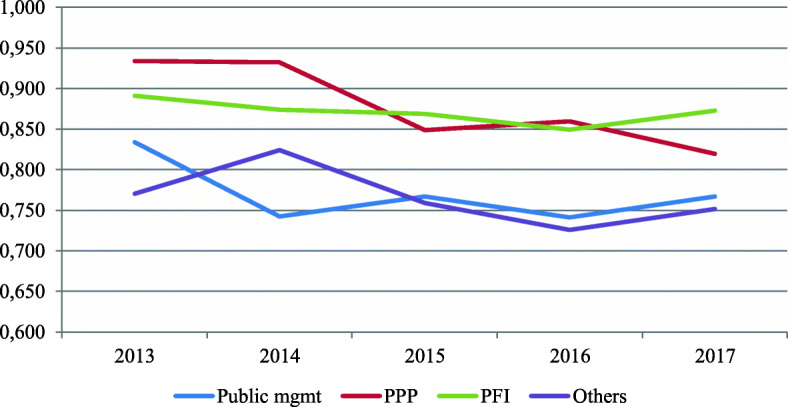
Evolution of unconditional efficiency scores for different types of management (2013–2017)

**Fig. 4:**
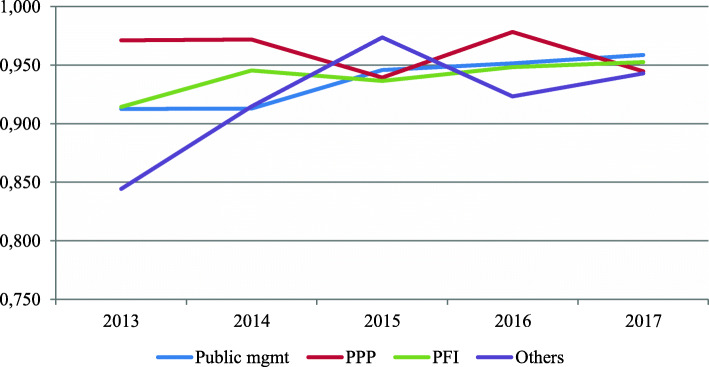
Evolution of conditional efficiency scores for different types of management (2013–2017)

If we examine the evolution of efficiency levels over the analyzed period, we can also find important divergences depending on whether we consider the context or not. Accordingly, Fig. 3 reveals that the unconditional efficiency estimates of all types of management have experienced a slight decrease over the studied period. Nevertheless, when we incorporate exogenous variables and the time factor into the estimation of conditional efficiency measures, there is an overall increase for all management models, except for hospitals managed by private initiatives (Fig. 4). One of the potential causes of this change in efficiency levels might be that traditional public hospitals are serving a higher proportion of the aging population (approximately 5%) than other forms of hospital management. Therefore, when we account for this information, the average efficiency of this type of hospital increases significantly.

In order to explore the influence of the contextual factors on efficiency estimates, we rely on the ratio between the conditional and the unconditional efficiency scores and the *p*-values of the significance tests explained in the previous section. The results suggest that all the variables have a significant impact on the hospitals´ performance, although the level of significance is relatively low for one of them (the morbidity index).

**Fig. 5:**
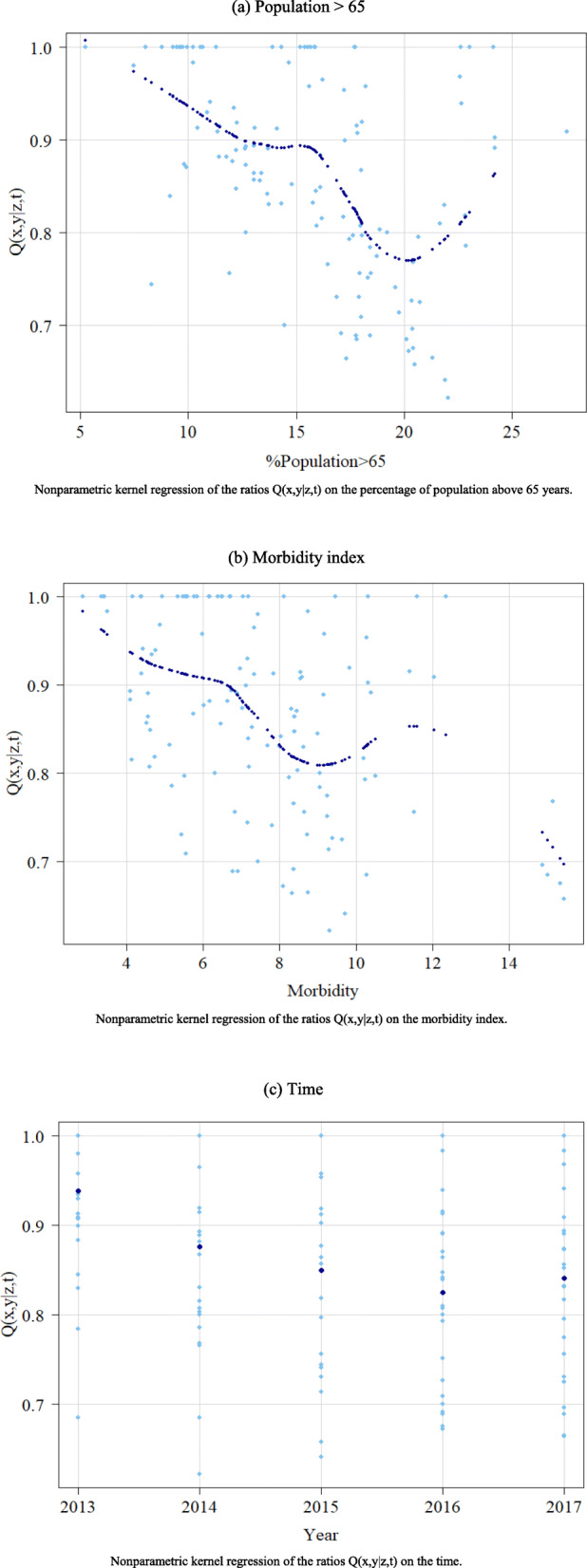
Marginal effect of each contextual variable on the ratios Q(x,y|z,t). **a** Population > 65. **b** Morbidity index. **c** Time

**Fig. 6:**
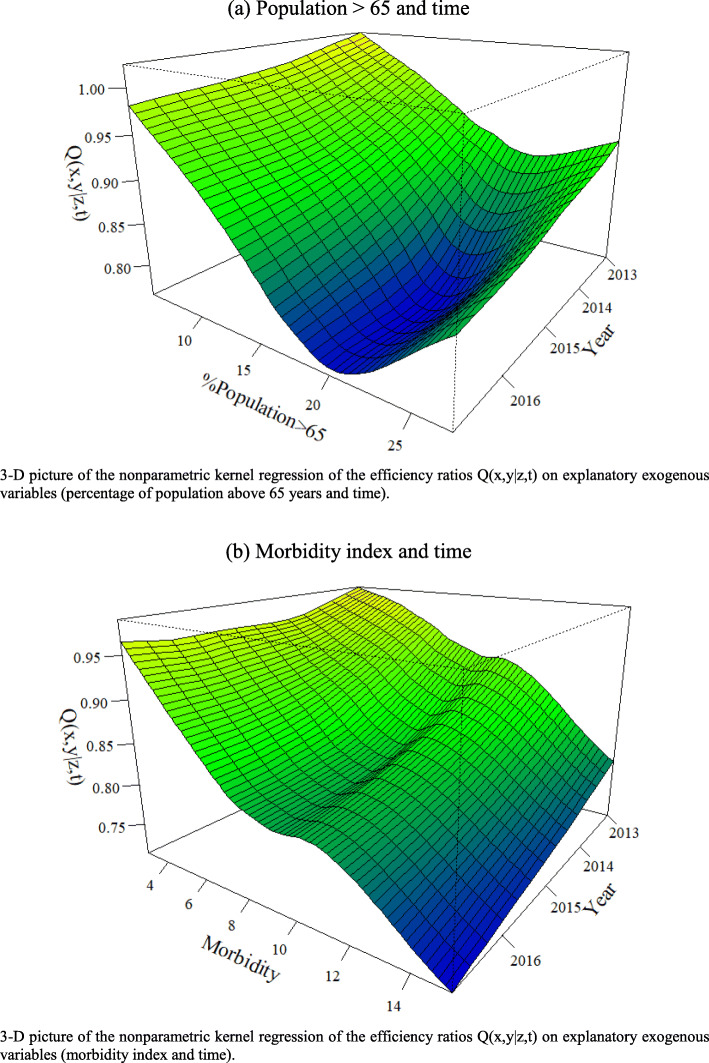
Effect of time and contextual variables on hospital performance: frontier shifts. **a** Population > 65 and time. **b** Morbidity index and time

With the aim of assessing the influence of contextual variables and time on hospital performance, we also analyze the ratios of conditional and unconditional efficiency measures against each Z. For this purpose, we examine the scatter plots reported in Fig. 5, which allows us to better visualize and interpret these effects. As we previously explained, since we adopt an output orientation, a decreasing nonparametric regression line indicates a negative effect, whereas an upward trend is associated with a favorable effect. In addition, Fig. 6 shows the three-dimensional pictures that illustrate the effects on the frontier (frontier shift) of contextual variables together with time, in which the interpretation of trends is analogous, but also allows us to visualize how the effect of variables evolves over the years.

According to the shape of the regression lines displayed in Fig. 5 a and b, the effect of both external factors seems to be unfavorable, although there is a small positive upturn for the highest values. This can be explained by the existence of a low number of units in the extreme of the distributions, which implies that most of them are considered as efficient in the conditional model. Likewise, we also observe negative effects for both variables in the 3D graphs (Fig. 6 a and b). This evidence aligns with previous studies focused on primary healthcare, where these contextual factors have also been found to be negatively associated with efficiency [14, 19]. Similarly, in Fig. 5 c, we also identify a slightly negative influence of time on the shift of the frontier, which suggests that the potential changes introduced in the healthcare system have led to a decrease in technological change.

## Discussion

Our empirical analysis shows that hospitals operating under new management models are more efficient in terms of chronic illness care coordination than traditionally managed hospitals, although their advantage over traditional models is much smaller when the operational environment is taken into account. The influence of a higher population above 65 years old in the areas covered by traditionally managed hospitals could be one of the main causes of this change in efficiency scores when environment is considered. Likewise, the morbidity index also presents lower values for PFI hospitals, which might explain their relatively better results in the initial analysis when the contextual factors are not taken into account. This evidence is partially consistent with other analyses of efficiency by management model type conducted in Spain, although they were focused on general outputs that are not related to integrated care [50, 51]. Note, conversely, that other studies did not find such a difference [11, 12].

The poorer coordination between care levels at public hospitals is striking, taking into account that primary care is a public monopoly; thus, we would expect that hospitals under the same ownership should be better coordinated. This result suggests that the efficiency of the new management models would be even better if primary care units were included. This is the case of the Valencian concession model, which has recently been found to achieve better results than the traditional public management [10].

For all the management models, we find important divergences across different units. The most relevant variability is detected for traditionally managed hospitals. Specifically, there is one hospital that clearly outperforms all the others (mean efficiency score of 0.99, clearly above the mean of 0.81 for all hospitals), while the remaining hospitals exhibit very low efficiency levels (mean efficiency of 0.64). Neither the profile of the population served nor the hospital size and service portfolio can account for these outlier values, as similar-sized hospitals with the same service portfolio achieve higher efficiency values.

Previous studies have shown that traditional public hospital resources are oversized with respect to state-owned enterprises or private hospitals [42]. Consequently, the desertion of traditional bureaucratic (governmental) practices enables more flexible and agile management [50, 51], along with increasing the professionalization of managers as opposed to the high level of politicization of high-ranking officers in public hospitals.

The results obtained show that public-private collaboration formulas are an efficient alternative to other existing management models. Likewise, we show that the variability between hospitals within each of the models suggests that the form and quality of management are more relevant than the management model as pointed out by Tsai et al. [52] and Lega et al. [53]. In any case, these more flexible forms of management are more accessible in public-private models than in traditional bureaucracy. Therefore, the results of our study seem to be relevant for both policymakers and healthcare managers, since they suggest strategies for improving health management and emphasize the need for the debate on health management policies and their reform options to be a process based on better evidence available. Moreover, having alternative management models allows for benchmarking (learning by comparison) and should be considered as an interesting contribution, particularly in contexts like Spain, where a return to more rigid administrative frameworks has been recently proposed.

One of the limitations of the methodology applied in our empirical analysis is the impossibility of establishing causal relationships, although this allows us to identify potential factors that might be affecting the level of efficiency, at least. Another potential limitation is that our analysis is conducted at the hospital level without including primary care, but the unified health district of the region of Madrid does not allow us to match each hospital with the primary care providers of reference as hospitals receive patients from all the primary care providers of the region. Additionally, we are aware that we should take into account that patients might change their reference hospital during the period due to the existence of a free choice system. Nevertheless, the low percentage of potential patients who exercise their right to freely choose a hospital (3.4% in 2016 and 3.8% in 2017) leads us to think that the adjustment for population characteristics is adequate, since there are no significant movements of potential patients between different hospital influence areas.

Finally, we need to be cautious when interpreting these results, bearing in mind that the period analyzed is relatively short and the number of hospitals that have adopted this management system is still limited.

## Conclusions

This is a groundbreaking study regarding the comparison of the technical efficiency of care coordination in the hospitals of the Region of Madrid operating under different management models. However, all of them have the same characteristics in terms of service portfolio and access guaranteed by the Spanish National Health System. Therefore, their differences in efficiency are not attributable to the characteristics of services provided but to the way in which service provision is managed.

This study has shown that public-private collaboration models are more efficient in the coordination of chronic illness care than conventional public management models and, therefore, those alternative management schemes should be considered as interesting alternative options by policy-makers in order to improve healthcare delivery. Improved efficiency could be attributed to hospital management being more agile and flexible under the collaborative models. Anyway, we should be cautious when extracting health policy lessons based on these findings as the analyzed period might be too short.

Moreover, the study reveals that there is room for improvement for most hospitals and a wide variability across units within each management model. This highlights not only the importance of the management model but also how management is exercised within each model.

Finally, the diversity of the results of this study gives all the managers of the hospitals of the Spanish Madrid region an opportunity for benchmarking and learning. Likewise, it is also an opportunity for policy-makers to place the debate on management models on technical aspects rather than on ideological perspectives.

## Supplementary Information

**Additional file 1.** [54].

## Data Availability

Further information on the datasets is available from the corresponding author on reasonable request.

## Abbreviations

SERMAS: Servicio Madrileño de Salud (Madrid Health Regional Service)
PFI: Private Finance Initiative
PPP: Public-Private Partnership
DEA: Data Envelopment Analysis
VRS: Variable Returns to Scale
COPD: Chronic Obstructive Pulmonary Disease
LSCV: Last Squares Cross-Validation
