# Supplementary material for: Efficiency in chronic illness care coordination: public-private collaboration models vs. traditional management
Source: BMC Health Serv Res. 2020 Nov 16;20:1044. doi: 10.1186/s12913-020-05894-z (PMC7667775; doi:10.1186/s12913-020-05894-z)
Supplement: Supplementary file 1 — Additional file 1. [54]. [file 12913_2020_5894_MOESM1_ESM.docx]

**APPENDIX**

The production technology used by production units (hospitals) to convert a set of inputs $x=\left( x_{1}, \ldots,x_{p} \right)\in R_{+}^{p}$ into a set of outputs $y=\left( y_{1}, \ldots,y_{q} \right)\in R_{+}^{q}$ can be defined as follows:

$\Psi=\left\{ (x,y)\in\mathbb{R}_{+}^{p+q}|x \text{can produce} y \right\}$ (1)

This production process can be defined by using an alternative probabilistic formulation. Following the notation introduced by Cazals et al. [37], the production process can be described by the joint probability function, denoted by $H_{X,Y}\left( x,y \right)$, which represents the probability of dominating a unit operating at level $\left( x,y \right)$:

$H_{X,Y}\left( x,y \right)=\text{Prob}\left( X\leq x,Y\geq y \right)$ (2)

This probability function can be further decomposed as follows:

$H_{X,Y}\left( x,y \right)=\text{Prob}\left( Y\geq y | X\leq x \right)\cdot\mathrm{Prob}\left( X\leq x \right)=$

$S_{Y|X}\left( Y\geq y | X\leq x \right)F_{X}\left( X\leq x \right)=S_{Y|X}\left( y | x \right)F_{X}\left( x \right)$ (3)

where$S_{Y|X}\left( y | x \right)$represents the conditional function of $Y$ and $F_{X}\left( x \right)$ represents the cumulative distribution function of $X$. Under free disposability and adopting an output orientation, the traditional measure of technical efficiency is given by

$\lambda(x,y)=\text{sup}\left\{ \lambda>0|H_{X,Y}(x,\lambda y)>0 \right\}=\text{sup}\left\{ \lambda>0|S_{Y|X}(\lambda y|x)>0 \right\}$ (4)

In this framework, it is possible to introduce contextual or environmental factors Z that might have an influence on the production process. Thus, the attainable conditional production set can be defined by

$\Psi^{Z}=\left\{ \left( x,y \right)\left| Z=z \right), x \text{can produce} y \right\}$ (5)

In the presence of these additional external factors, the conditional distribution can be defined using a probabilistic model that conditions the production process to certain values of these variables ($Z=z$):

$H_{XY\left| Z \right.}(x,y\left| z)=Pr(X\leq x,Y\geq y\left| Z=z) \right. \right.$ (6)

This function represents the probability of a unit at the given level ($x,y$) of being dominated by other units facing the same environmental conditions $Z=z$. F This can also be decomposed into two terms: the survival conditional function of outputs ($S_{Y|X,Z}(y|x,z)$) and the conditional distribution function of inputs ($F_{X|Z}(x|z)$). Therefore, the output efficiency measure can be analogously defined as:

$\lambda\left( x,y | z \right)=\text{sup}\left\{ \lambda>0|H_{X,Y|Z}\left( x,\lambda y | z \right)>0 \right\}=\text{sup}\left\{ \lambda>0|S_{Y|X,Z}(\lambda y|x,z)>0 \right\}$ (7)

As our analysis aims to assess efficiency over a period of time, we need to extend this model to a dynamic framework including the time dimension. Following Mastromarco and Simar [18], we consider time factor ($t$) as an additional conditional variable; thus, we have the following set of production possibilities:

$H_{\left. X,Y \right|Z}^{t}\left( \left. x,y \right|z \right)=Prob\left( \left. X\leq x,Y\geq y \right|Z=z,T=t \right)=S_{Y|X,Z}^{t}\left( y | x,z \right)F_{X|Z}^{t}\left( x | z \right)$ (8)

The conditional output-oriented efficiency measures can be defined as:

$\lambda^{t}(x,y|z)=sup\left\{ \lambda| H_{XY|Z}^{t}(x,\lambda y|z)>0 \right\}=sup\left\{ \lambda\left| S_{Y|XZ}^{t}\left( \lambda y\left| x,z \right. \right)>0 \right. \right\}$ (9)

By a plug-in rule, different nonparametric estimators can be used to estimate the total frontier $\hat{\lambda^{t}}\left( x,y|z \right)$. In this work, we adopt the well-known DEA alternative following the formulation proposed by Daraio and Simar [17], which implies a frontier built from real and fictitious units since we are assuming a convex technology.

The computation of conditional efficiency estimators requires adopting smoothing techniques. For that purpose, we apply the approach proposed by Badin et al. [38] for bandwidth selection (*h*):

$\hat{S}_{\left. Y \right|X,Z}^{t}\left( \left. y \right|x,z \right)=\frac{\sum_{j=\left( i,v \right)} I\left( x_{j}\leq x,y_{j}\geq y \right)K_{h_{z}}\left( z_{j}-z \right)K_{h_{t}}\left( v-t \right)}{\sum_{j=\left( i,v \right)} I\left( x_{j}\leq x \right)K_{h_{z}}\left( z_{j}-z \right)K_{h_{t}}\left( v-t \right)}$ (10)

Here $h_{z}$ and $h_{t}$ are the bandwidth of optimal size and $K(\cdot)$ is a kernel function with compact support. For this study, optimal bandwidths are selected using the least squares cross-validation (LSCV) procedure suggested by Li and Racine [54]. This approach has the appealing feature of detecting the irrelevant factors and smoothing them out by providing large bandwidth parameters.
